# Atrial Fibrillation and Risk of Dementia: Epidemiology, Mechanisms, and Effect of Anticoagulation

**DOI:** 10.3389/fnins.2019.00018

**Published:** 2019-01-31

**Authors:** Rose Mary Ferreira Lisboa Da Silva, Cláudia Madeira Miranda, Tong Liu, Gary Tse, Leonardo Roever

**Affiliations:** ^1^Department of Internal Medicine, Federal Institute of Minas Gerais, Belo Horizonte, Brazil; ^2^Department of Cardiology, Tianjin Cardiovascular Institute, Tianjin Chest Hospital, Tianjin, China; ^3^Department of Medicine and Therapeutics, The University of Hong Kong, Pokfulam, Hong Kong; ^4^Department of Clinical Research, Federal University of Uberlandia, Uberlândia, Brazil

**Keywords:** atrial fibrillation, dementia, cognition, Alzheimer's disease, anticoagulation therapy

## Abstract

Atrial fibrillation (AF) is one of the cardiovascular risk factors for dementia. Several longitudinal studies have reported an association between AF and dementia independently of stroke history. Although the mechanisms underlying this association are not fully understood, proposed mechanisms include cerebral hypoperfusion, inflammation, genetic factors, cerebral microbleeds, and recurrent silent cerebral ischemia. Oral anticoagulation can be used to minimize risk of cognitive decline and dementia, given that brain insults can be caused by chronic microemboli or microbleeds. However, controversy on the effects of warfarin and direct oral anticoagulants on this risk exists. This article will address these aspects, with data on the studies already published and a critical view on this subject.

## Introduction

### Epidemiological Data

Dementia can present with typical signs such as memory deficits and executive dysfunction, interfering with daily life activities or atypical signs including pronounced clinical problems (language, visual, practic, or executive problems) before memory impairment. The main cause is Alzheimer's disease (accounting for up to 75%) and the majority of patients are older more than 65 years (Moschetti et al., 2012; Qiu and Fratiglioni, 2015; Scheltens et al., 2016). With the improvement of health care diseases with target organ damage, such as the brain, vascular dementia occurs in 10–20% of cases. It is estimated an increased prevalence of dementia, especially in developing countries, where the population is younger than that of developed countries (Scheltens et al., 2016). There was a 117% increase in the number of individuals with dementia in the period between 1990 and 2016, with the aging population and its growth. There is a pre-dominance in women and dementia is the fifth cause of death in the world, with socioeconomic impact (GBD 2016 Disease Injury Incidence Prevalence Collaborators., 2017).

The description of dementia dates back to 1906, when a clinical and neuroanatomist psychiatrist, Alois Alzheimer, reported “A peculiar process of severe cerebral cortex disease” in a 50-years-old woman, who had progressive sleep and memory disorders, aggression and confusion, evolving to death 5 years later. Distinct plaques and neurofibrillary tangles were observed in the histology of her brain. In 1910, Kraepelin, the Alzheimer's mentor, included “Alzheimer's Disease” in the eighth edition of his text Psychiatrie (Hippius and Neundörfer, 2003). There are many well-recognized risk factors for dementia. Those that are modifiable include diet, low educational attainment, physical and mental inactivity, obesity, smoking, air pollution, hypertension, diabetes, higher total serum cholesterol, cardiovascular diseases, which altogether may account for 30% of the risk of dementia. By contrast, non-modifiable risk factors include advancing age, female, black ethnicity, and genetic factors (Norton et al., 2014; Dagres et al., 2018; Ganguli et al., 2018).

Of the different cardiovascular diseases, atrial fibrillation (AF) is a risk factor independently of stroke history (Tse et al., 2017). AF has been demonstrated by the electrocardiogram for more than a century. It is an arrhythmia with a great public health burden, constituting a worldwide epidemic. Its prevalence increases with age, being approximately 3% in those with at least 20 years of age, reaching more than 13% in the elderly over 80 years. It is estimated that its current prevalence will increase from 8.8 million to around 18 million by 2060 (Rahman et al., 2014; Kirchhof et al., 2016).

However, the first description of the relationship between AF and dementia was made in a post-mortem case series of 48 patients with dementia, of which 47% presented history of AF (Ratcliffe and Wilcock, 1985). This was subsequently confirmed by the Rotterdam Study in 1997 (Ott et al., 1997). Since these seminal reports, several longitudinal studies have reported a significant association between AF and dementia (Jacobs et al., 2015; Dagres et al., 2018). This may partly be due to the common risk factors underlying both diseases, such as advanced age, physical inactivity, alcohol consumption, hypertension, diabetes, heart failure, vascular disease, renal failure, sleep apnea, and genetic factor (Jacobs et al., 2015; Kirchhof et al., 2016).

## Mechanisms of Atrial Fibrillation Leading to Dementia

Advanced age and systemic vascular risks contribute to an atrial cardiomyopathic process with constant abnormal structural and electrophysiological remodeling, predisposing to AF (He et al., 2017; Tse et al., 2018a,b). Late life dementia coexists with many other neuropathological processes, such as Lewy bodies, neurofibrillary tangles, and hippocampal sclerosis; an additional factor such as AF would accelerate these pathological processes, reducing cognitive reserve (Kamel et al., 2016).

Although the mechanisms between AF and dementia are not fully understood, the main mechanisms involved have been reduced and intermittent cerebral perfusion during arrhythmia or silent cerebral ischemia caused by thromboembolism and inflammatory biomarkers. The beat-to-beat variations with reduced cardiac output present in AF rhythm may result in transient or chronic cerebral hypoperfusion. This hypothesis is supported by evidence of low and high ventricular rate response as predictors of dementia and a higher prevalence of cognitive impairment in patients with heart failure and AF (Jacobs et al., 2015; Rivard and Khairy, 2017).

Stroke and subclinical infarcts (evidenced by imaging methods) due to hypercoagulable state, circulatory stasis and endothelial injury may explain the association of AF and multi-infarct dementia (Jacobs et al., 2015). The most vulnerable area for developing cerebral microbleeds is the hippocampus, which is also the area in which the damage is frequent in patients with Alzheimer's disease and AF. However, the relationship between dementia and AF can occur independently of stroke.

The proinflammatory state is implicated in the genesis and perpetuation of AF. In turn, inflammation potentiates hypercoagulability and thrombus formation, predisposing to stroke. The inflammatory markers identified were C-reactive protein, interleukin (IL) IL-2, IL-6, and IL-8, tumor necrosis factor alpha, among others (Dietzel et al., 2018).

Genetic factors have been studied and AF-related gene (PITX2) was significantly associated with dementia. These markers may help explain the paradoxical higher relative risk of dementia in younger patients with AF (Jacobs et al., 2015; Rivard and Khairy, 2017).

## Trials on Atrial Fibrillation as a Risk Factor For Dementia

There is evidence of the association between dementia and AF demonstrated by prospective and retrospective observational studies, as well as by cross-sectional studies and by systematic reviews and meta-analyzes.

### Observational Studies

Previous longitudinal studies have included 377–37,025 participants, mean age between 61 and 88 years, clinical follow-up of 2.2 years (mean) over 25 years, with hazard ratio of up 2.61 (Jacobs et al., 2015). For diagnosis of dementia, Mini-Mental State Examination (MMSE) and cognitive test were performed. For example, a study of 37,025 patients without dementia and a follow-up of at least 5 years showed that patients younger than 70 years of age had the highest relative risk of developing Alzheimer's dementia among those with AF (Bunch et al., 2010). This shows that the association between dementia and AF is more than an epiphenomenon due to pathological conditions that share old age as a common risk factor. Cognitive decline was also observed among 10% of 5,150 participants who had no history of AF at the beginning of follow-up and who had AF over 7 years (Thacker et al., 2013). This demonstrates the interaction of two hands between the two clinical conditions.

The link between AF and dementia appears to be independent of cerebral vascular involvement. In a community cohort of 2,837 patients diagnosed with AF without cognitive dysfunction, or stroke at baseline, with follow-up for a mean period of 4.5 years showed that there was a relationship between the new dementia detection and arrhythmia independent of stroke (Miyasaka et al., 2007). Another prospective population-based Rotterdam-study with 6,514 participants free of dementia reported higher risk of dementia among those < 67 years of age and who had long duration of AF (de Bruijn et al., 2015). Cohort study of 332,665 AF patients without dementia demonstrated a dementia risk ratio of 1.42 for a follow-up between 1996 and 2011 after adjusting for age, gender, use of medication, and baseline differences (Liao et al., 2015).

The Framingham Heart Study, a prospective cohort, single-site, community-based study with 2,682 participants, investigated the association of AF and cognitive decline using domain specific neuropsychological test performance. Participants were free of dementia and stroke at baseline and were underwent at least one additional test after 1 year. There was a significant association between AF with worse performance in executive function tests, particularly in men. In addition, patients with AF had a significant longitudinal decline in executive function, when compared to those without AF (Nishtala et al., 2018). Moreover, the Atherosclerosis Risk in Communities (ARIC) study, with 12,515 participants, found that 2,106 participants developed AF and 1,157 participants developed dementia during a 20-years follow-up. Participants who developed AF had greater cognitive decline after adjustment for ischemic stroke. Furthermore, the incidence of AF was associated with a 23% higher risk of dementia (Chen et al., 2018).

The major cross-sectional studies on the association between FA and mild cognitive impairment and/or dementia are summarized in Table 1, and the main prospective and retrospective studies in Table 2.

**Table 1:** Main cross-sectional studies.

**Study (year)**	**Number of patients**	**Age (years)**	**Diagnosis screening of dementia or CI**	**Results**
Ott et al. (1997)	6,584 (195 with AF)	69.2	MMSE score < 26	Positive associations of AF with both dementia (odds ratio 2.3) and CI (1.7)
Jozwiak et al. (2006)	2,314 (hospitalized)	76	MMSE score < 24	Positive association of AF with CI (odds radio 1.56)
Elias et al. (2006)	1,011 men (59 with AF)	61	Neuropsychological testing	Significantly lower mean levels of cognitive performance in men with AF.
Kawabata-Yoshihara et al. (2012)	1,524 (37 with AF)	> 65	DSM-IV criteria	Odds ratio for dementia in participants with AF was 2.8
Di Nisio et al. (2015)	784 (103 with AF)		DSM-IV	AF was associated with 2.0-fold increase in vascular dementia and 1.72-fold increase in AD
Alonso et al. (2017)	6,432 (611 with AF)	76 (no AF); 79 (with AF)	Neurocognitive battery	AF was associated with increased odds of dementia (2.25) and CI (1.28).

*Age, mean or limit; CI, cognitive impairment; AF, atrial fibrillation; MMSE, mini-mental state examination; AD, Alzheimer's disease; DSM, diagnostic and statistical manual of mental disorders*.

**Table 2:** Main prospective and retrospective studies.

**Study (year), design**	***N***	**Age (years)**	**Diagnosis screening of dementia or CI**	**Follow-up (years)**	**Results**
Tilvis et al. (2004), prospective	650	>75	MMSE and CDR	up 10	Five-years decline was predicted by AF (risk of 2.8)
Forti et al. (2007), prospective	611	75.2	Neurocognitive battery and MMSE	3.8	AF associated with dementia with a risk of 4.63 among those with mild cognitive impairment
Peters et al. (2009), prospective	3,336	≥80	longitudinal MMSE scores	2	No relationship of AF with annual change in MMSE (multivariate analysis)
Bunch et al. (2010), prospective	37,025	60.6	ICD-9 codes	5	AF was independently associated with all forms of dementia
Dublin et al. (2011), prospective	3,045	74.3	Cognitive abilities screening instrument	6.8	AF was associated with a 40 to 50% higher risk of both AD and all-cause dementia, independent of stroke.
Marengoni et al. (2011), prospective	685	>75	MMSE	6	AF was not associated with dementia
Haring et al. (2013), prospective	6,455 women	65–79	MMSE and neurocognitive examination	8.4	No significant association between AF and CI
Marzona et al. (2012), prospective	31,506	66.5	MMSE	4.66	AF was associated with an increased risk of CI (hazard ratio 1.14) and new dementia (1.30)
Thacker et al. (2013), prospective	5,150	73	MMSE	7	AF was associated with CI in the absence of clinical stroke
Rusanen et al. (2014), prospective	1,510	65–79	ICD and DSM-IV	7.8	AF in late-life was an independent risk factor for dementia (risk 2.610
de Bruijn et al. (2015), longitudinal community-based study	6,514	68.3 without AF; 75.7 with AF	DSM-III	20	AF was associated with an increased risk of dementia, independent of clinical stroke
Liao et al. (2015), longitudinal community-based study	332,665	70.3	ICD	14	AF was significantly associated with the occurrence of dementia (risk 1.42)
Marzona et al. (2016), retrospective	1,600,200 (without AF); 27,431 (hospitalized for AF)	75.2 (without AF); 78.4 (with AF)	ICD	10	AF was associated with a higher risk of dementia (17%)
Singh-Manoux et al. (2017), prospective	10,538 (for analysis of incident dementia)	45–85	Serial battery of cognitive tests	26.6	AF had 87% excess risk of dementia
Nishtala et al. (2018), cross-sectionally and longitudinally	2,682	72	Neurocognitive battery	6	AF was significantly associated with CI
Chen et al. (2018), prospective	12,515	56.9	Cognitive tests	20	AF was associated with an increased risk of dementia (risk 1.23) independent of ischemic stroke

*N, number of patients; CI, cognitive impairment; MMSE, mini-mental state examination; CDR, clinical dementia rating; AF, atrial fibrillation; RR, relative risk; ICD, International classification of diseases; AD, Alzheimer's disease; DSM, diagnostic and statistical manual of mental disorders*.

### Review and Meta-Analysis Studies

There are systematic reviews and meta-analyzes to assess the association between AF and dementia. A systematic review of three cross-sectional, two case-control and three prospective studies, with direct comparison with patients in the control group in sinus rhythm, reported the association between cognitive impairment and AF. Patients with AF had a risk of 1.7–3.3 of cognitive impairment and a 2.3 risk of dementia when compared to patients without AF (Udompanich et al., 2013).

Three meta-analyses with prospective and cross-sectional studies, including 46,637; 77,668 and 85,770 patients, also demonstrated that there is a risk of dementia in those with AF, with or without a history of stroke (Kwok et al., 2011; Santangeli et al., 2012; Kalantarian et al., 2013). The hazard ratios ranged from 1.4 to 2.3 for the risk of dementia and from 1.7 to 3.3 for cognitive decline. However, the risk of dementia and cognitive decline is more modest in those without stroke at baseline than in patients with AF and previous history of stroke. In conclusion, several longitudinal and cross-sectional studies have demonstrated the link between AF and cognitive impairment and dementia. This positive association also remained significant in multivariate analysis and there was a strong association between AF and dementia in patients younger than 70–75 years old.

However, there are limitations on these studies as the diagnostic criteria for cognitive impairment and the method of detecting AF. It is necessary to distinguish between dementia and mild cognitive impairment. In dementia, there is severe acquired cognitive impairment interfering with social and/or occupational life. When everyday activities are performed independently, yet with modest difficulty in one or more cognitive domains, there is a state of mild cognitive impairment (Hugo and Ganguli, 2014). Although cross-sectional studies allow to verify the association between AF and dementia, they fail to provide information on the temporal sequence of these conditions. In addition, the diagnosis time and the classification of the AF as well as the adherence to the treatment are not always clarified.

## Effect of Oral Anticoagulation

Different interventions may be useful for targeting AF through rate or rhythm control, or reduce systemic inflammation such as statins, as well as anticoagulation. Although there is little evidence on the effect of anticoagulation on silent cerebral infarctions and the risk of cognitive decline, delayed use of warfarin or its misuse relative to international normalized ratio increases the risk of dementia (Dagres et al., 2018). Among the hypotheses about the association between AF and dementia there is the brain injury caused by chronic microembolism or microbleeds. The use of oral anticoagulant demonstrated a 60% reduction in the risk of dementia in a study with 2,685 participants without dementia, mean age 73.1 years, during which 11.4% had AF and 14.9% presented dementia during the follow-up period 9 years (Ding et al., 2018). There is also an influence on the quality of anticoagulation. A study of 2,605 patients, mean age of 73.7 years, median follow-up of 4 years (maximum 9.9 years) showed that 109 had dementia. Those with dementia were older and had a higher CHADS_2_ score, in addition to lower mean percent of time in therapeutic range (TTR). After adjustment, as a continuous and categorical variable, TTR was associated with an increased risk of dementia, with a hazard ratio of up to 5.34, comparing TTR >75% with TTR equal to or < 25% (Jacobs et al., 2014).

However, there is interdependence between cognitive function and adequate anticoagulation. A low score in the MMSE is an independent predictor of an international normalized ratio out of range. In a study of 2,510 patients with a mean age of 71 years from 27 countries, for each 1-point decline in MMSE baseline score between 30 and 25, there was a 1-point reduction in TTR. Moreover, the reduced MMSE scores were associated with an increased risk of bleeding and vascular events during follow-up of 1.3 years (Flaker et al., 2010).

By contrast, a recent systematic review of 19 studies published up to November 2014 including 15,876 participants, demonstrated a modest protective effect of oral anticoagulation, however without decreasing the rate of dementia in patients with AF (Moffitt et al., 2016). The authors of this review commented on the substantial risks of bias, such as sample size, follow-up time, cognitive decline by other mechanism, inclusion of patients using antiplatelet agents, among others.

With a larger population and a more appropriate selection, a more recent meta-analysis with of 471,057 participants with AF and under oral anticoagulation demonstrated that anticoagulation was associated with a significant reduction in cognitive impairment. Moreover, comparing non-vitamin K oral anticoagulants (NOAC) and warfarin, NOAC group significantly reduced the occurrence of dementia and there was increased risk of bleeding with warfarin (Cheng et al., 2018). Therefore, brain microhemorrhages may have implications in the mechanism of dementia. Anticoagulation in the supratherapeutic range has been associated with the risk of dementia in patients with AF. Furthermore, patients taking NOAC had a low combined risk of dementia and stroke (Madhavan et al., 2018).

Another major meta-analysis, including studies published through February 2018, including 13,484,202 patients with AF, demonstrated that vitamin K antagonists reduced the risk of dementia and cognitive decline by 23% when compared to the group without oral anticoagulation. Despite the influence of the highest TTR on that reduction, the strength of evidence was low (Mongkhon et al., 2018).

As a result of these controversies, blind and randomized studies are important to verify the role of oral anticoagulation in the prevention of dementia. One ongoing study is the Blinded Randomized Trial of Anticoagulation to Prevent Ischemic Stroke and Neurocognitive Impairment in AF (BRAIN-AF) (NCT02387229). Participants with non-valvular AF will be submitted to MMSE and other tests to rule out dementia prior to randomization. The efficacy and safety of rivaroxaban 15 mg with matching acetylsalicylic acid—placebo or acetylsalicylic acid 100 mg with matching rivaroxaban-placebo will be compared for stroke reduction, transient ischemic attack and neurocognitive decline. There is another study titled “Impact of Anticoagulation Therapy on the Cognitive Decline and Dementia in Patients With Non-Valvular Atrial Fibrillation (CAF)” randomized, which will compare the use of dabigatran and warfarin in 120 patients to assess the cognitive decline through neurological examination and cognitive testing. The first 10 patients from each treatment group will be submitted to cranial magnetic resonance at baseline and at 24 months post-anticoagulation (US National Library of Medicine, 2017a).

## Current Research Gaps

In addition to the controversies previously mentioned, there are other gaps in this field of knowledge, such as the type of cognitive test used for the diagnosis of dementia, the longitudinal association and the presentation of AF with specific types of dementia. The influence of treatment on cognitive impairment is another knowledge gap. There is a risk of bleeding with the use of oral anticoagulants. Therefore, there was the advent of percutaneous exclusion of the left atrial appendage as a method to reduce stroke in patients who have contraindication to anticoagulation (Chanda and Reilly, 2017). Due to complications related to the procedure and the operator's experience, this field is still under development. However, there are no studies demonstrating about these devices and the risk of dementia.

Atrial fibrillation catheter ablation in a population-based propensity-to-match score reduced stroke and mortality risk (Saliba et al., 2017). However, the non-ablation group was older, with a higher proportion of arterial hypertension, vascular disease and heart failure, while the ablation group had a higher proportion of patients taking rhythm control medications, beta-blocker, and anticoagulant medications. The effect on the cognitive function of catheter ablation is unknown although ablation is associated with additional silent brain lesions. Neurocognitive dysfunction may occur, with a prevalence of 13–20% within 90 days after the procedure (Medi et al., 2013). There are no published studies on the influence of this treatment on reducing the incidence of cognitive decline or dementia. There is a randomized trial in progress with the secondary objective of assessing the effects of ablation and antiarrhythmic on cognitive function (US National Library of Medicine, 2017b).

## Potential Future Developments

Given that there are many factors in common between AF and dementia, the interrelationships between these conditions are not yet fully elucidated and more studies are needed. Large prospective multicenter studies are also required to examine the impact of warfarin, direct oral anticoagulants, statins, rhythm and rate control, and left atrial appendage occlusion on incidence and progression of dementia. These trials should be randomized controlled with long follow-up, including comprehensive neurocognitive assessment and brain imaging. Another aspect not yet investigated is the influence of brain changes associated with cognitive decline in the risk of AF.

## Conclusion

AF and dementia are clinical conditions with similar risk factors with an age-dependent increase in prevalence. Mechanisms between the two conditions are not fully understood and may be multifactorial independently of stroke. Anticoagulation may be effective to reduce risk of developing dementia.

## Author Contributions

RS, CM, GT, TL, and LR analyzed and interpreted the data, provided important technical and intellectual contents, conceived, designed, and oversaw this study, drafted the manuscript. All the authors revised and approved the final manuscript.

### Conflict of Interest Statement

The authors declare that the research was conducted in the absence of any commercial or financial relationships that could be construed as a potential conflict of interest.
